# Distribution and Conservation of *Ephedra rhytidosperma*

**DOI:** 10.1002/ece3.70762

**Published:** 2024-12-27

**Authors:** Chao Tan, David Kay Ferguson, Yong Yang

**Affiliations:** ^1^ Co‐Innovation Center for Sustainable Forestry in Southern China, State Key Laboratory of Tree Genetics and Breeding, College of Life Sciences Nanjing Forestry University Nanjing China; ^2^ Department of Paleontology University of Vienna Vienna Austria

**Keywords:** conservation gap, distribution patterns, *Ephedra rhytidosperma*, in situ, MaxEnt

## Abstract

With global warming and increasingly intensified human activities, numerous species are on the verge of extinction, ca. 28% of living species are threatened globally, although conservation of endangered species has received worldwide attention. It remains unclear if threatened species have been appropriately conserved or not. *Ephedra rhytidosperma* is an endangered species and included in the List of National Key Protected Wild Plants in China (released in September 2021). This shrubby species is endemic to the Helan Mountains in northwestern China where it dominates the lowland vegetation. We have conducted an integrative investigation on the conservation of the species. We used the MaxEnt model to predict the potential geographic distribution of *E. rhytidosperma* under past, current, and future climatic scenarios based on distributional occurrences and environmental data and investigated the conservation status and its effectiveness. The results show that *E. rhytidosperma* is mainly distributed in lowland Helan Mountains, while the range in the past and future show different patterns. The range has shrunk significantly and migrated westwards since the Last Interglacial, whereas the projected area in the future displays a fluctuating pattern and easterly migration. The precipitation (Bio14), the temperature (Bio9), and degree of slope (Slope) are the dominant factors influencing its current and future ranges. We also found that *E. rhytidosperma* populations at different altitudes showed different adaptations to the environment. Our assessment of the conservation status of the hotspots revealed that only 15.1% occur in nature reserves, implying that a large conservation gap remains. In addition, there has been less attention paid to *ex situ* conservation. As a result, we propose conducting an integrative conservation approach including both *in situ* and *ex situ* management to save *E. rhytidosperma* . Our study lays a solid foundation for the development of targeted conservation strategies for *E. rhytidosperma* .

## Graphical abstract

In this manuscript, we investigated and assembled distribution occurrences of *E. rhytidosperma* , and conducted analyses including (1) potential geographic distribution of *E. rhytidosperma* under different climatic scenarios and dominant environmental factors, (2) different environmental adaptations at different altitudes, and (3) evaluated the conservation effectiveness in nature reserves and proposed targeted conservation actions.

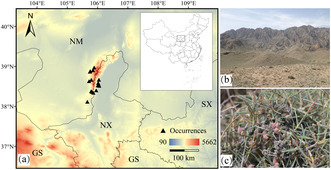

## 1 Introduction

Climate is one of the most important factors shaping species distribution patterns, community structure, and composition, as well as functions of the ecosystem and landscape configuration (Scholze et al. 2006; Bellard et al. 2012; Vermeiren, Reichert, and Schuwirth 2020). During the Quaternary, repeated oscillations of glacial and interglacial periods occurred on a global scale, with a series of climatic fluctuation events experienced worldwide since the Last Interglacial (LIG, 140–120 ka BP) in particular (Chen, Kang, and Liu 2011). These strongly influenced the past population dynamics of species and shaped the present‐day patterns of plant distributions. However, the global climate is becoming progressively warmer due to intensified human activities since the industrial revolution (Klein and Anderegg 2021), which has led to the diminution of species on the globe (Powers and Jetz 2019; Chase et al. 2020; Richards, Thompson, and Wijedasa 2020). As a result, it is necessary to focus on the potential distribution patterns of species under different climatic scenarios in order to develop effective species conservation strategies.

The maximum entropy (MaxEnt) model is one of the powerful tools to explain the distribution pattern of species and is widely used in studying the distribution of endangered species under climate change, management of invasive species, conservation biology, and evolutionary biology (Gonzalez‐Hernandez et al. 2020; Saputra and Han 2021; Campos‐Soldini 2022; Liu et al. 2022). The model is based on existing species distribution records and environmental data, and is highly compatible with presence/absence data and even a small amount of existing data; this model has the advantages of high accuracy, small sample size requirement, good stability, and is usually superior to other models (Fitzpatrick, Gotelli, and Ellison 2013; Merow, Smith, and Silander 2013; Morales, Fernández, and Baca‐González 2017; Wu, Wang, and Mu 2022). Currently, the proportion of research in which MaxEnt modeling predicts species' potential ranges is increasing and is applicable to different community types (Wang et al. 2023). Analyses based on the MaxEnt model can provide a clear understanding of the distribution of endangered species under different climatic scenarios and also lay a theoretical basis for conservation strategies of species.

Helan Mountains, the border between Ningxia and Nei Mongol, are the transitional region between the temperate grassland and desert regions in northwestern China, and harbor relatively high plant species richness and vegetation diversity (Li and Zhang 2014). *Ephedra rhytidosperma*, belongs to the family Ephedraceae, is a dwarf shrub, endemic to the Helan Mountains in China, and is mainly found at intermediate altitudes in these landscapes (Tao et al. 2001; Yang 2007). Climate change and intensified human activities have led to the destruction of the habitat of *E. rhytidosperma*, so its distribution range has been shrinking, resulting in a sharp decrease in populations (Zhang et al. 2006). The species was assessed as endangered (EN, Yang 2021), and has been included in the updated List of National Key Protected Wild Plants (NKPWP, http://www.forestry.gov.cn/main/5461/20210908/162515850572900.html/). However, studies of this species are very rare, for example, phylogeny (Wang et al. 2005), micromorphology and development (Yang 2007); community ecology (Shi et al. 2022); and localized geographic patterns (Zhao et al. 2022), and a comprehensive conservation study of the species is lacking. Species distribution modeling based on the distribution data of *E. rhytidosperma* has become crucial for recognizing and understanding suitable distribution areas, as well as rationally formulating conservation strategies.

To explore the potential response of *E. rhytidosperma* to current and future climate change, we collected and screened distributional data and relevant environmental data of this species. Then, we used the MaxEnt model to simulate the potential geographic distribution of this species based on past, present, and future climatic scenarios and analyzed the differences in the potential distribution areas and spatial dynamics. Finally, we assessed the distribution of hotspots in nature reserves under current and future climatic scenarios, analyzed the gaps, and made conservation recommendations including *in situ* and *ex situ* management.

## 2 Materials and Methods

### 2.1 Collection and Processing of Occurrence Data

**FIGURE 1:**
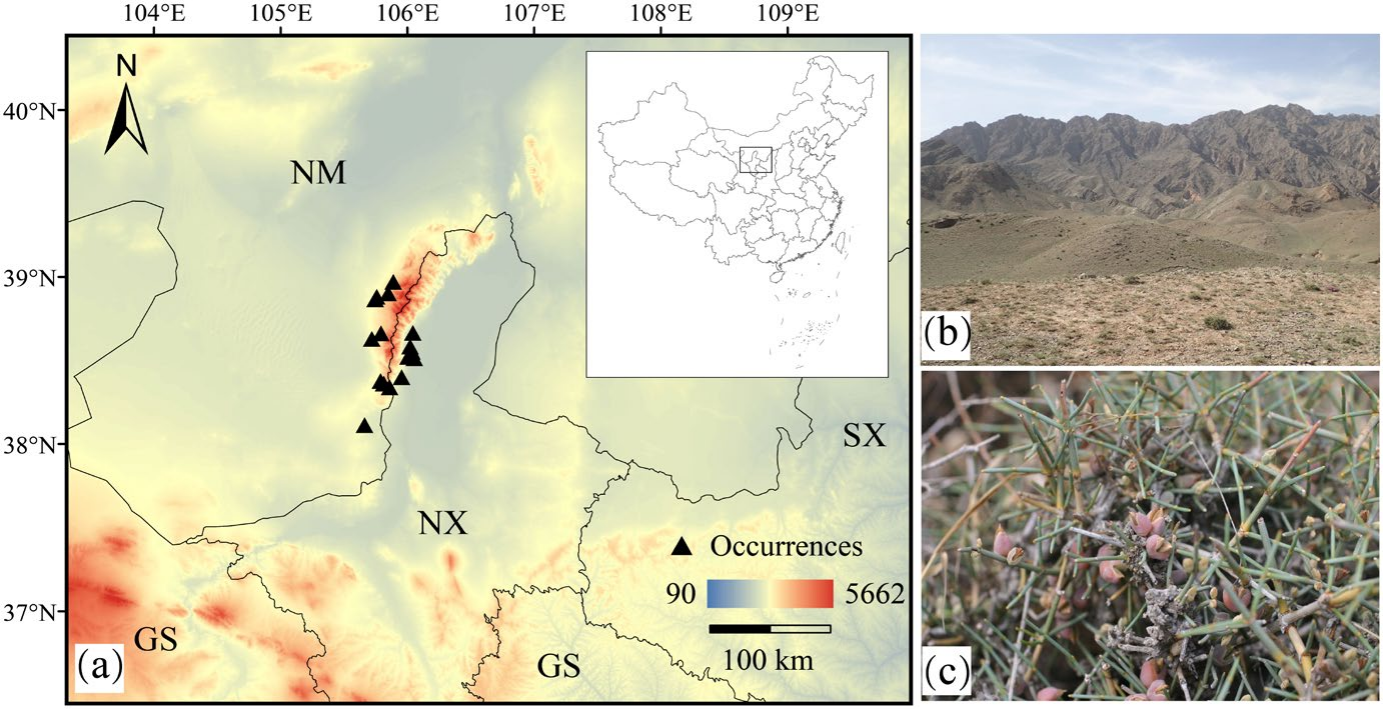
Distribution of *E. rhytidosperma* . (a) distribution areas. GS, Gansu; NM, Nei Mongol; NX, Ningxia; SX, Shaanxi; (b) Community; (c) Morphology.

The distribution data of *E. rhytidosperma* originated from two main sources: (1) our research group conducted field surveys and collected 42 distribution records of natural populations in Nei Mongol and Ningxia; (2) 13 and 11 distribution records were retrieved through the Chinese Digital Herbarium (CVH, https://www.cvh.ac.cn/) and National Herbarium Information Infrastructure (NSII, http://www.nsii.org.cn/), respectively. We excluded some distributional anomalies and cultivated records based on the natural distributions of *E. rhytidosperma* , and retained only the native distributional occurrences. Considering the high degree of redundancy between different datasets, we deleted the duplicate occurrence records prior to MaxEnt analysis and imported the distribution data into ArcGIS 10.2 to eliminate the duplicates, that is, we retained only one of the distribution points within 10 km^2^ (Zhou et al. 2021). Finally, we obtained 16 valid distribution records of *E. rhytidosperma* , including eight in Nei Mongol on the western slope of the Helan Mountains and eight in Ningxia on the eastern slope of the Helan Mountains (Figure 1, Table 1).

**TABLE 1:** Dataset of *E. rhytidosperma* used in this paper.

Locality	Longitude	Latitude	Elevation (m)
Alxa Zuoqi, Nei Mongol	105.7294500	38.8743830	1692
Alxa Zuoqi, Nei Mongol	105.8704830	38.9787330	1774
Alxa Zuoqi, Nei Mongol	105.6406110	38.1241670	1424
Alxa Zuoqi, Nei Mongol	105.7653656	38.3876915	1673
Alxa Zuoqi, Nei Mongol	105.7405396	38.8920822	1685
Alxa Zuoqi, Nei Mongol	105.8315811	38.9104195	1858
Alxa Zuoqi, Nei Mongol	105.7005386	38.6381798	1538
Alxa Zuoqi, Nei Mongol	105.7720108	38.6710587	1790
Xixia District, Yinchuan City, Ningxia	105.9800000	38.5900000	1229
Xixia District, Yinchuan City, Ningxia	106.0100000	38.5600000	1183
Xixia District, Yinchuan City, Ningxia	105.7897415	38.3714790	1777
Xixia District, Yinchuan City, Ningxia	105.8376236	38.3488350	1551
Xixia District, Yinchuan City, Ningxia	106.0282898	38.5218658	1150
Xixia District, Yinchuan City, Ningxia	105.9941330	38.5914078	1205
Xixia District, Yinchuan City, Ningxia	106.0181046	38.6725349	1202
Xixia District, Yinchuan City, Ningxia	105.9281670	38.4080670	1293

### 2.2 Environment Variables Acquisition and Screening

To explain the distribution characteristics of *E. rhytidosperma* , 32 environmental factors were included in this study (Table 2). Nineteen climate variables (Bio1–Bio19) and elevation parameters for the current climate were downloaded from WorldClim (Fick and Hijmans 2017). Since the climate in July and August significantly affects seed germination in *E. rhytidosperma* (Zhang and Tao 2015), we downloaded 10 climate factors from WorldClim for the two months. Degree and direction of slope were extracted from the altitude using ArcGIS 10.2. In addition, paleoclimatic data of three stages, namely, LIG (*~*120,000–140,000 years BP), Last Glacial Maximum (LGM; *~*22,000 years BP), and Middle Holocene (Mid‐Holocene, MIH; *~*6000 years BP), were downloaded from PaleoClim (http://www.paleoclim.org/). We used the climate model BCC‐CSM2‐MR to predict the potential distribution of *E. rhytidosperma* under three shared socioeconomic pathways (SSP126, SSP245, and SSP585) in the future (2050, 2070) (Sang et al. 2021). Additionally, future monthly temperatures and precipitation were downloaded from the Resource and Environment Science and Data Center of the Chinese Academy of Sciences (https://www.resdc.cn/). The spatial resolution of the variable layers selected for this paper was all 2.5′.

**TABLE 2:** Environmental variables examined in this study and the percentage contribution of selected variables to the current distribution of *E. rhytidosperma.* The 10 most important variables are marked in bold.

Variables	Description	Units	Percent contribution (%)
Bio1	Annual mean temperature	°C	—
Bio2	Mean diurnal range	°C	—
Bio3	Isothermality (bio2/bio7) × (100)	—	—
**Bio4**	Temperature seasonality (standard deviation × 100)	°C	8
Bio5	Max temperature of warmest month	°C	—
Bio6	Min temperature of coldest month	°C	—
Bio7	Temperature annual range (bio5–bio6)	°C	—
Bio8	Mean temperature of wettest quarter	°C	—
**Bio9**	Mean temperature of driest quarter	°C	28.6
Bio10	Mean temperature of warmest quarter	°C	—
Bio11	Mean temperature of coldest quarter	°C	—
Bio12	Annual precipitation	mm	—
Bio13	Precipitation of wettest month	mm	—
**Bio14**	Precipitation of driest month	mm	23.6
Bio15	Precipitation seasonality (coefficient of variation)	—	—
Bio16	Precipitation of wettest quarter	mm	—
Bio17	Precipitation of driest quarter	mm	—
Bio18	Precipitation of warmest quarter	mm	—
Bio19	Precipitation of coldest quarter	mm	—
ELE	Elevation	m	—
Aspect	Direction of slope	—	—
**Slope**	Degree of slope	°	18.5
Preci07	Monthly precipitation in July	mm	—
**Preci08**	Monthly precipitation in August	mm	7.1
Tavg07	Monthly mean temperature in July	°C	—
Tavg08	Monthly mean temperature in August	°C	—
Vapr07	Monthly vapor pressure in July	kPa	—
Vapr08	Monthly vapor pressure in August	kPa	—
Srad07	Monthly solar radiation in July	KJ/m^2^/d	—
**Srad08**	Monthly solar radiation in August	KJ/m^2^/d	14.2
Wind07	Monthly wind speed in July	m/s	—
Wind08	Monthly wind speed in August	m/s	—

Climate layers were cropped according to the study area using the software ArcGIS 10.2 and converted to ASCII format. In order to avoid overlap between environmental factors that could lead to unreliable simulation results (Luo et al. 2017), we performed Pearson correlation analyses of the climatic variables using the “cor” function of the R software, retaining the climatic factors with higher contributions to the model at *r* < |0.7| (Zhou et al. 2021), and finally selecting six variables to be included in the analyses for the current, past and future climatic scenarios (Table 2). In addition, we conducted a principal component analysis (PCA) to determine the variability of drivers in different distribution areas of *E. rhytidosperma* populations.

### 2.3 MaxEnt Analysis

As fewer than 25 valid recording points were available for *E. rhytidosperma* , we used the Jackknife method for simulation evaluation, that is, when constructing the model, one of the coordinates was removed, and the remaining *n*‐1 coordinates were used to build the model, so that n models could be built, with the optimal model being selected for the simulation of MaxEnt ecological niches (Pearson et al. 2007; Zhou et al. 2021), while default values were used for the remaining parameters. The accuracy of the model was evaluated according to the area under curve (AUC) value: AUC < 0.6 indicates model failure, 0.6 < AUC < 0.7 indicates poor reliability, 0.7 < AUC < 0.8 indicates moderate reliability, while AUC > 0.9 indicates high reliability (Swets 1988). Next, the MaxEnt model simulation results were imported into ArcGIS 10.2 software and transformed into raster layers for visualization. Based on the correlation between the survival probability of *E. rhytidosperma* and the dominant variables, we took the fitness index *p* < 0.25 as an unsuitable area, 0.25 < *p* < 0.5 as a barely suitable area, 0.5 < *p* < 0.75 as moderately suitable area, and *p* > 0.75 as a highly suitable area. We used *p* > 0.75 as the hotspot area and calculated the total range, the hotspot areas, and proportion under future climatic scenarios.

To compare variation in the range of *E. rhytidosperma* in past, present, and future climatic scenarios, we first overlapped the hotspot area of the past and current periods to determine the stable distributional region (Xu et al. 2017; Tang et al. 2022). Second, we characterized the centroid movement of *E. rhytidosperma* distribution and calculated future migration distances by combining longitude and latitude (Yan et al. 2021). Finally, we used the standard deviation ellipse (SDE) to analyze the dynamics of the suitable range under current and future climatic scenarios (Lefever 1926).

### 2.4 Area Calculation of Hotspots in Nature Reserves

To assess the conservation status of *E. rhytidosperma* in Chinese nature reserves, we collected data on nature reserves established since 1956. By the end of 2017, China had 2750 nature reserves, accounting for 14.9% of the total land area (MEP 2019). In our study, the database of nature reserves (NRs) includes 440 national nature reserves (NNRs) and more than 800 provincial nature reserves (PNRs) (Zhang et al. 2015). Subsequently, in ArcGIS 10.2, we overlapped layers of Chinese nature reserves with hotspots to calculate the area located in NRs under current and future climatic scenarios and assess their conservation efficiency. In addition, we calculated the extent of occurrence (EOO) and area of occupancy (AOO) of *E. rhytidosperma* using coordinate data in GeoCAT (http://geocat.kew.org/) and re‐assessed its current threatened status. Finally, we assessed the endangered status of *E. rhytidosperma* under future climate change based on A3(c) of the Guidelines for Using the IUCN Red List Categories and Criteria (IUCN Standards and Petitions Subcommittee 2024).

## 3 Results

Based on the final distribution and environmental data, the potential range of *E. rhytidosperma* was simulated using the optimal MaxEnt model. The results show that the AUC values of the simulated curves are > 0.9 for each time period scenario, indicating that the predictions of the model are very reliable (Table 3).

**TABLE 3:** Contribution of the main variables in different scenarios (Unit: %).

Variables	LIG	LGM	MIH	Current	2050 ssp126	2050 ssp245	2050 ssp585	2070 ssp126	2070 ssp245	2070 ssp585
Bio9	15.6	47	33.1	28.6	30.2	30.1	32.1	29.9	32.9	29.4
Bio14	15.4	15.9	24.5	23.6	24.1	24.5	23	23.8	22.1	25
Srad08	17.1	10	10.6	14.2	13.1	12.1	10.4	11	11.8	12.3
Slope	18.9	12.5	14.1	18.5	20.5	21.4	23	20.9	21.7	21.6
Bio4	25.6	8.6	10.6	8	5.4	6.9	4.4	6.8	3.8	4.6
Preci08	7.3	6	7	7.1	6.8	5.1	7.1	7.7	7.7	7.1
AUC	> 0.9	> 0.9	> 0.9	> 0.9	> 0.9	> 0.9	> 0.9	> 0.9	> 0.9	> 0.9

### 3.1 Changes in the Past and Current Distribution Patterns of *Ephedra rhytidosperma*

**FIGURE 2:**
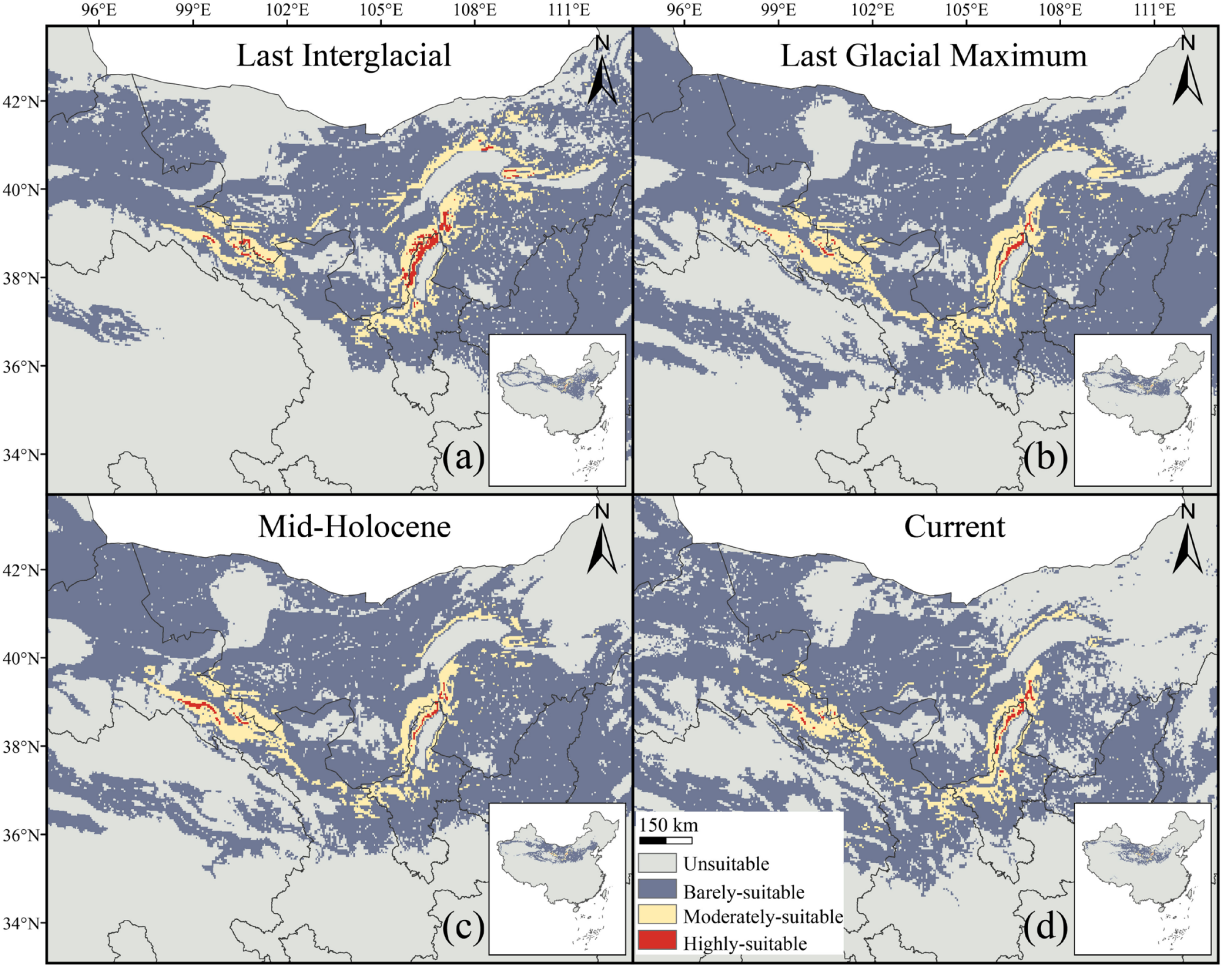
Potential distribution patterns of *E. rhytidosperma* under past and current scenarios.

Based on the model prediction results, the range of *E. rhytidosperma* in the past and current climate scenarios showed significant variation, with an overall contraction trend (Figure 2). This species occurred in a wide range of habitats in the LIG, with multiple distribution centers extending from Liaoning to Xinjiang (Figure 2a). As a result of paleoclimatic changes, *E. rhytidosperma* gradually became limited to the central part of Nei Mongol, north‐central Ningxia, and Gansu, with a small number of outliers in Xinjiang, Qinghai, Shaanxi, Shanxi, Hebei, and Tibet (Figure 2b–d).

**FIGURE 3:**
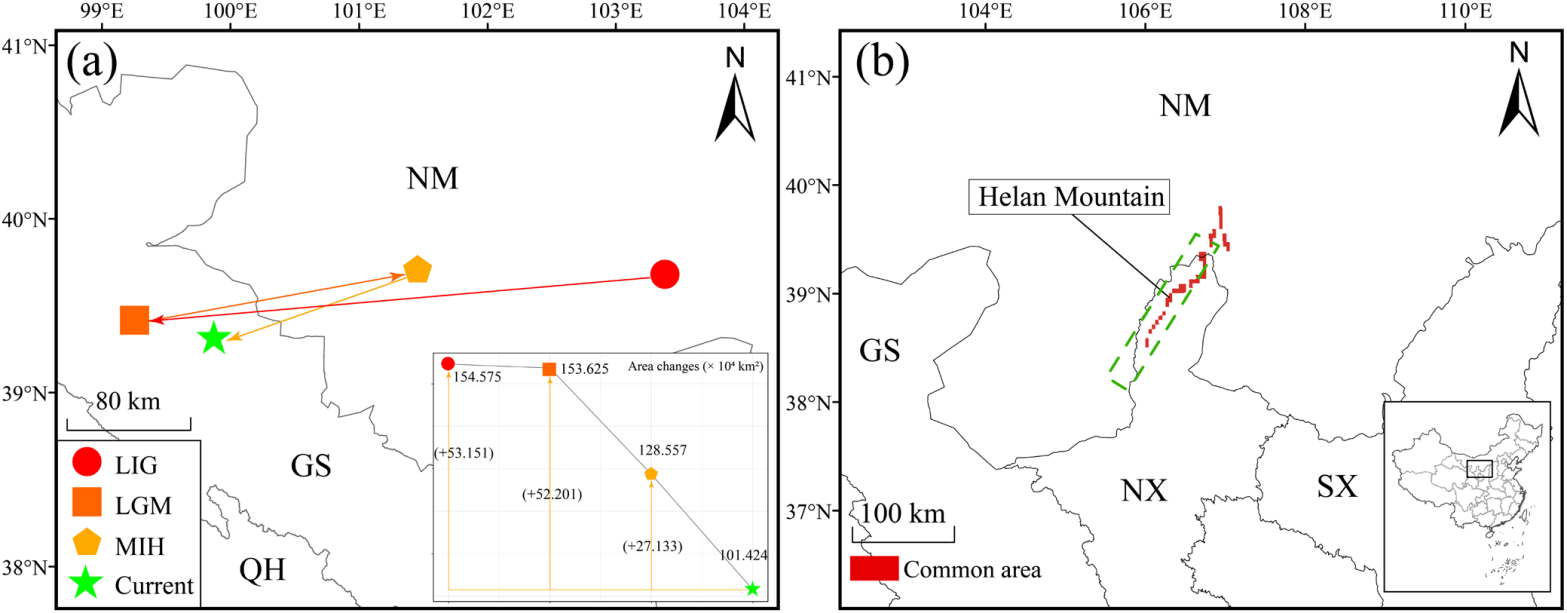
Trends in the distribution area of *E. rhytidosperma* . (a) Relocation routes and area changes. (GS, Gansu; NM, Nei Mongol; QH, Qinghai); (b) Common areas of overlap between past and present periods (SX, Shaanxi).

Model predictions based on the past and current environments show that the range of *E. rhytidosperma* has been significantly decreasing since the LIG, with an overall decrease of 53.151 × 10^4^ km^2^, which is equivalent to about half of the current total range (Figure 3a). The greatest decrease in the range was 27.133 × 10^4^ km^2^ between the Mid‐Holocene and the present, while the minimum decrease was 9.5 × 10^3^ km^2^ between the LIG and the LGM (Figure 3a). In addition, the range of *E. rhytidosperma* has generally migrated westwards over a wide region, except for an easterly movement during the LGM (Figure 3a). By overlaying the ranges of the four past and current time periods, we found that *E. rhytidosperma* was mainly distributed in the Helan Mountains and its fringes, with an area of 8.681 × 10^2^ km^2^ (Figure 3b).

### 3.2 Changes in the Future Distribution Patterns of *E. rhytidosperma*

**FIGURE 4:**
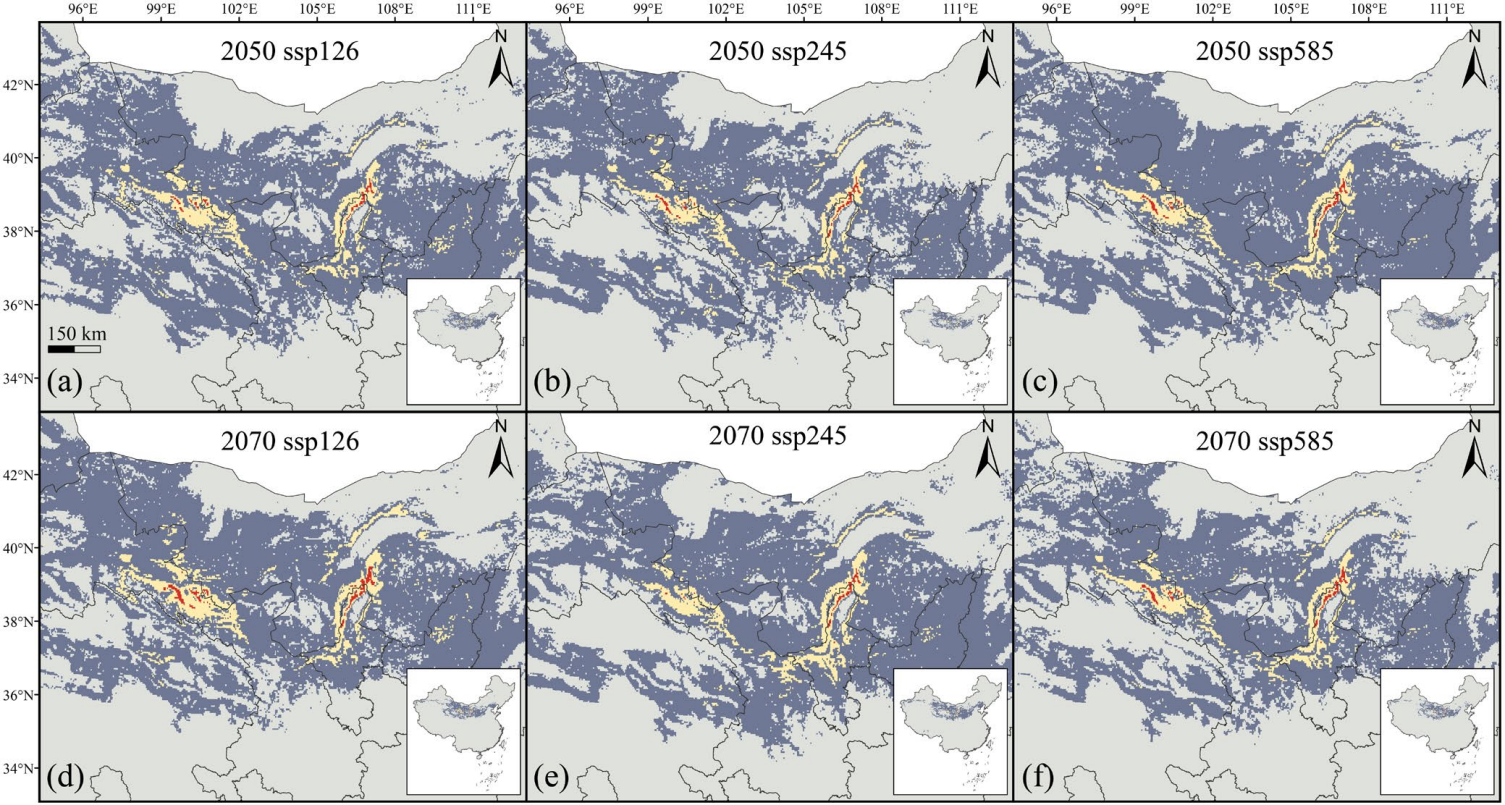
Potential distribution patterns of *E. rhytidosperma* under future climate scenarios.

According to the model results, the overall changes in the range of *E. rhytidosperma* under various future climate scenarios were insignificant compared with the current situation, being mainly concentrated in the central part of Nei Mongol, the north‐central part of Gansu, and the Helan Mountain region on the border between Nei Mongol and Ningxia, with a small amount in Xinjiang, Qinghai, Shaanxi, Shanxi, but almost absent from the rest of the area (Figure 4).

**FIGURE 5:**
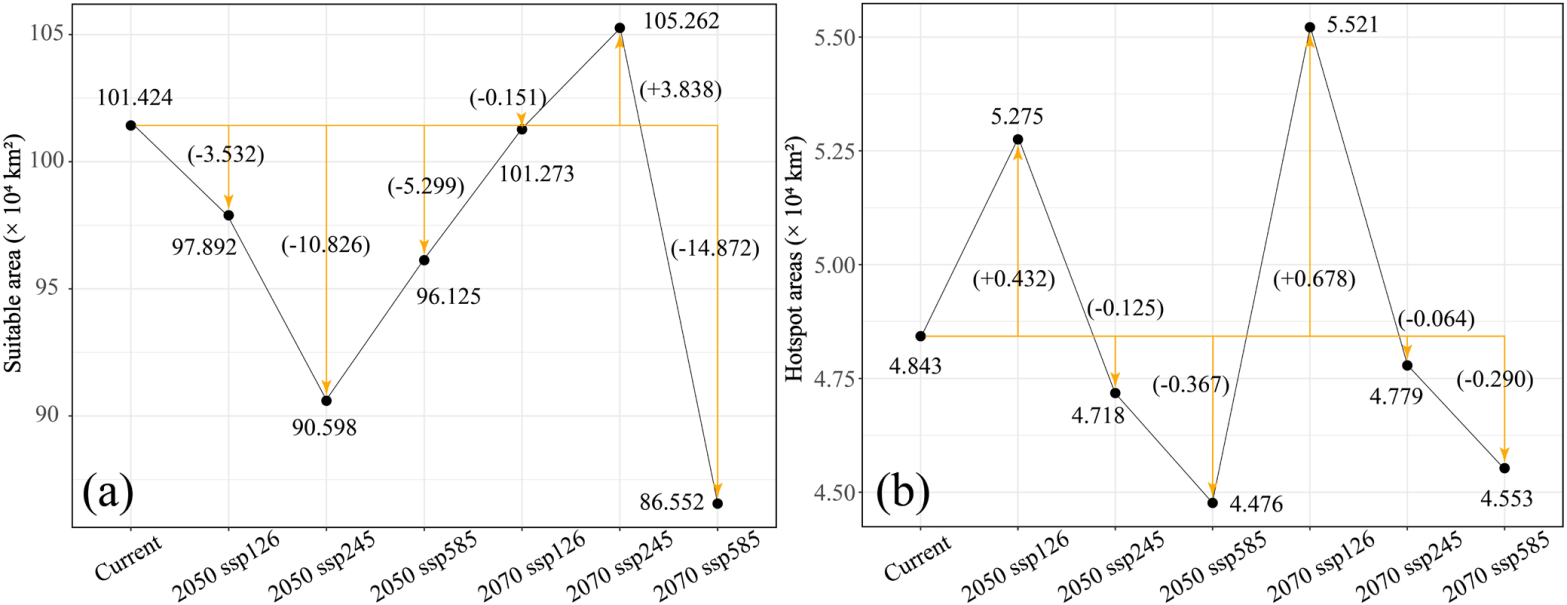
Expansion and contraction trends in the suitable areas of *E. rhytidosperma* under different climatic scenarios. (a) Suitable areas; (b) Hotspot areas.

The model‐based predicted distribution for *E. rhytidosperma* shows increasing and decreasing trends under future climate change. The total range at present is 101.424 × 10^4^ km^2^. Regarding future climate changes, it shows an overall decreasing trend compared to the present, except for the 2070 ssp245 climatic scenario where the potential range increases by 3.838 × 10^4^ km^2^ (Figure 5a). Of these, the 2070 ssp585 climatic scenario displays the most severe reduction in range, that is, 14.872 × 10^4^ km^2^. The least shrinkage occurs in the 2070 ssp126 climatic scenario, viz. 1.51 × 10^3^ km^2^. The rest of the future climatic scenarios display contractions of 3.532 × 10^4^, 10.826 × 10^4^, 5.299 × 10^4^ km^2^, respectively, in that order (Figure 5a).

### 3.3 Center of Distribution Movement of *E. rhytidosperma* Under Future Climatic Scenarios

**FIGURE 6:**
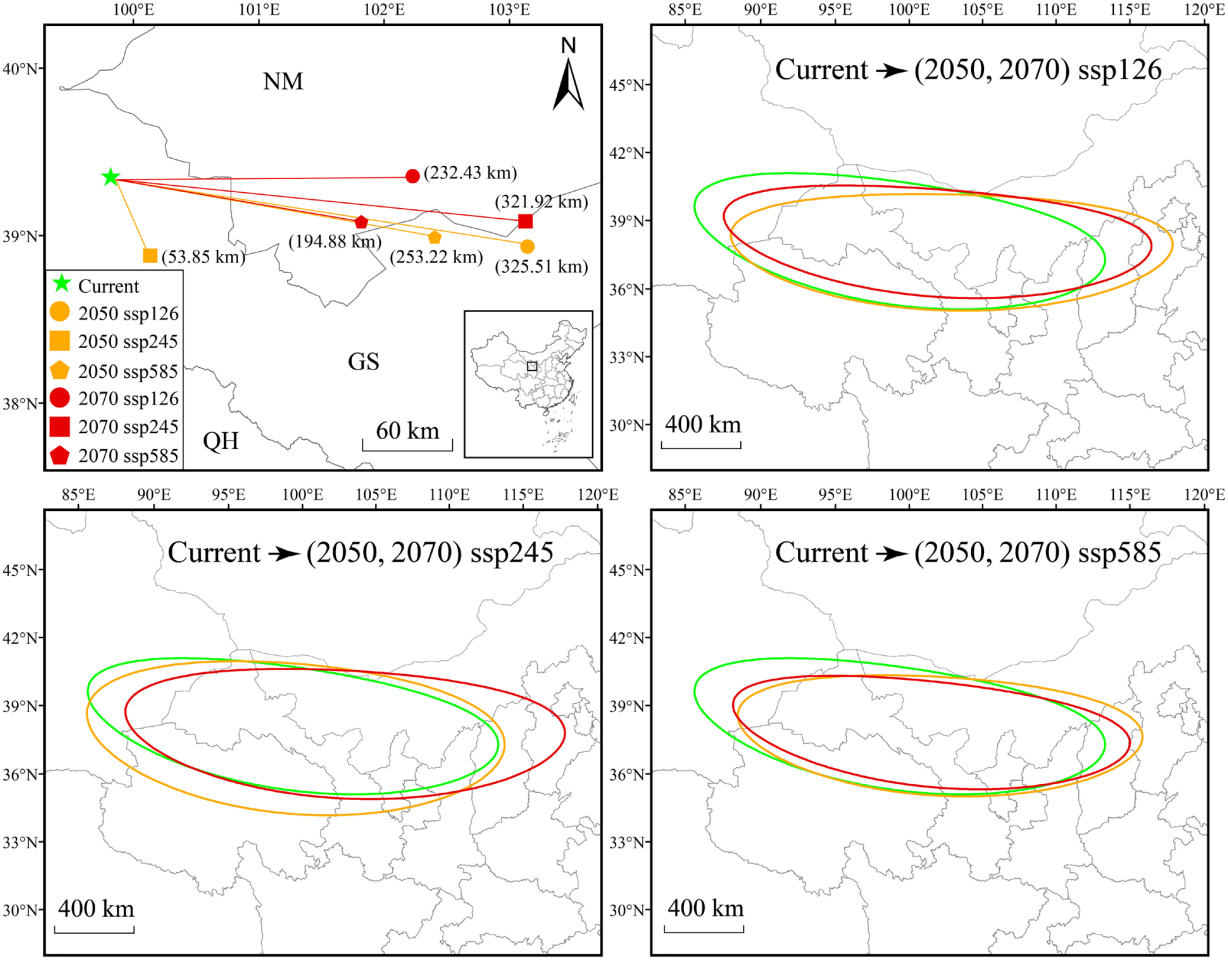
The standard deviational ellipses and centers of migrations of *E. rhytidosperma* under different climatic scenarios.

Overall easterly migration of the center of distribution of *E. rhytidosperma* is expected under future climatic scenarios (Figure 6a). Moreover, all the centroids migrate in a southeasterly direction. In the 2050 ssp126 climatic scenario, the population centroid should move the furthest in a southeasterly direction, covering a distance of 325.51 km, while the smallest distance occurs in the 2050 ssp245 climatic scenario, shifting only 53.85 km in a northwesterly direction. SDE analyses confirm that the center of distribution of *E. rhytidosperma* will shift in an overall southeasterly direction under all climatic conditions (Figure 6b–d).

### 3.4 Distribution of Hotspots and Conservation Status of *E. rhytidosperma* Under Future Climatic Scenarios

**FIGURE 7:**
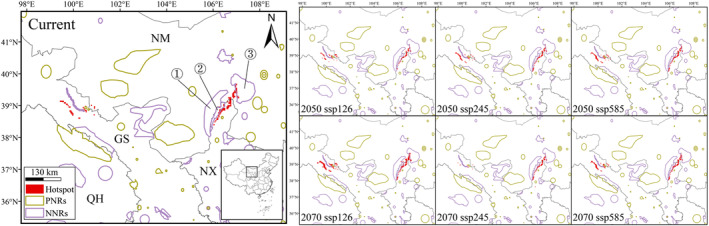
Hotspot areas located in national nature reserves (NNRs) and provincial nature reserves (PNRs) of *E. rhytidosperma* under different climatic scenarios. ([1] Helan Mountain NNR of Nei Mongol; [2] Helan Mountain NNR of Ningxia; [3] West Ordos NNR).

Under future climatic scenarios, the hotspots of *E. rhytidosperma* will remain mainly concentrated in the Helan Mountains, with some areas in north‐central Gansu and central Nei Mongol, but basically no occurrences in the rest of the region (Figure 7). However, the predicted hotspot areas showed various expanding and contracting trends in the natural area (Figure 5b). Two of the climatic scenarios (2050 ssp126, 2070 ssp126) display expansion, while the remaining four climatic scenarios (2050 ssp245, 2050 ssp585, 2070 ssp245, and 2070 ssp585) show a reduction in range. The hotspot area of *E. rhytidosperma* is 4.843 × 10^4^ km^2^ under the current climate. The highest hotspot area occurs in the 2070 ssp126 climate scenario, reaching 5.521 × 10^4^ km^2^, an increase of 6.78 × 10^3^ km^2^. The smallest range is expected to occur in the 2050 ssp585 climatic scenario, that is, 4.476 × 10^4^ km^2^, a decrease of 3.67 × 10^3^ km^2^. Regarding the rest of the future climates, hotspot areas are 5.275 × 10^4^, 4.718 × 10^4^, 4.779 × 10^4^, and 4.553 × 10^4^ km^2^, respectively (Figure 5b).

The area of *E. rhytidosperma* in the nature reserves (NRs) is low under all climatic scenarios and does not exceed 16% (Figure 7, Table 4). Only 15.1% of the hotspot area is located in NRs under the current climate, mainly in the Helan Mountain NNR and the West Ordos NNR (Figure 7). The conservation effectiveness of the hotspot areas shows a decreasing trend under all future climate conditions, with only 8.66% in 2050 ssp245. While the 2070 ssp126 climatic scenario has the highest hotspot area in NRs and NNRs, the 2050 ssp126 climatic scenario registers the lowest value in NRs and NNRs (Table 4).

**TABLE 4:** Hotspot areas and proportion of NRs under different climatic scenarios (Unit: Km^2^).

Period	Hotspot areas	Hotspot areas in NRs			Proportion
		NNRs	PNRs	Total	
Current	2205.21	330.54	2.38	332.92	15.10
2050 26	1840.57	163.66	0.01	163.67	8.89
2050 45	2257.31	190.66	4.89	195.55	8.66
2050 85	2500.4	241.22	22.31	263.53	10.54
2070 26	3837.42	437.13	13.4	450.53	11.74
2070 45	1545.39	201.21	0.02	201.23	13.02
2070 85	2760.86	316.39	23.78	340.17	12.32

*Notes:* Proportion = Total areas/Hotspot areas.

### 3.5 Differences in Environmental Adaptation of *E. rhytidosperma* in Different Regions

**FIGURE 8:**
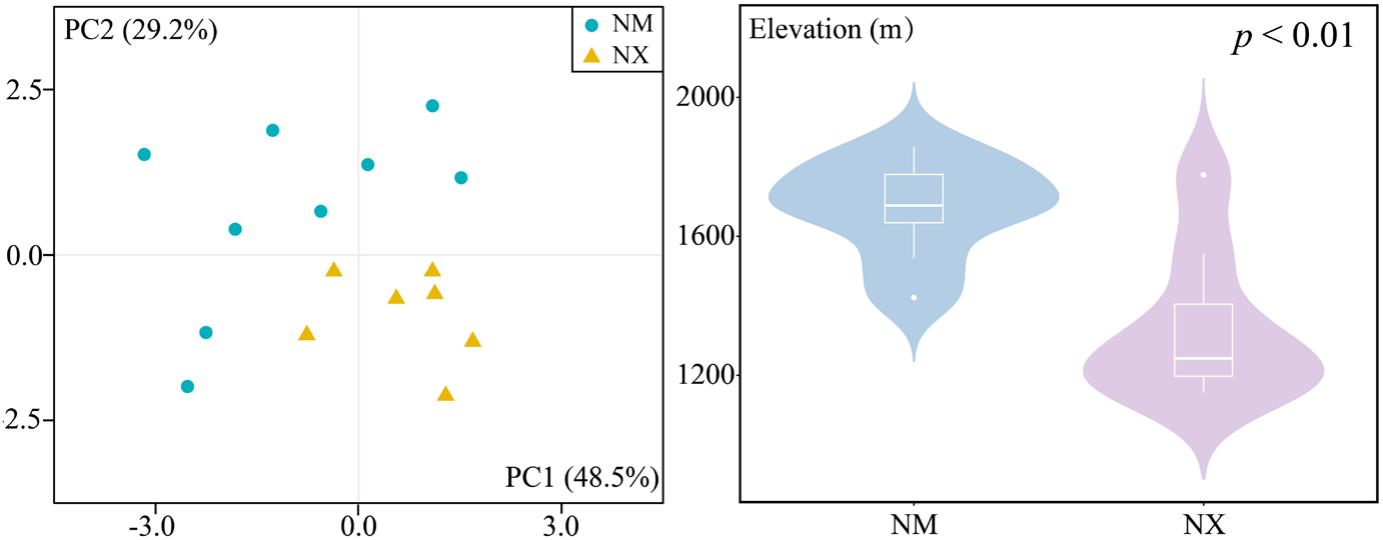
Comparison of distributional variability of *E. rhytidosperma* . (a) PCA; (b) Elevation. (NM, Nei Mongol; NX, Ningxia).

Populations of *E. rhytidosperma* in Nei Mongol and Ningxia show great differences in environmental adaptation. In this study, positive values on the horizontal axis of PCA (PC1) indicate climates characterized by a gradual decrease in precipitation of the Driest Month (Bio14), while positive values on the vertical axis (PC2) indicate climates with large temperature seasonal variations (Bio4) (Figure 8a, Table 5). As a result, populations in Ningxia tend to grow at lower altitudes where there is less precipitation and lower seasonal temperature variations, while populations in Nei Mongol occur at middle altitudes with relatively higher precipitation and seasonal temperature variations (Figure 8).

**TABLE 5:** Contribution of environmental variables and cumulative variability in PCA analysis.

Variables	PC1	PC2	PC3
Bio4	0.321	0.917	−0.111
Bio9	0.739	−0.562	0.058
Bio14	−0.942	0.419	−0.285
Srad08	0.722	0.101	0.195
Preci08	−0.818	−0.563	0.045
Slope	−0.427	0.308	0.844
Cum. (%)	48.49	77.71	91.87

## 4 Discussion

### 4.1 Distributional Variability and Dominant Variables of *E. rhytidosperma*

Previous studies have shown that the abundant precipitation and favorable temperatures in the mid‐elevation region of the Helan Mountains (1800–2500 m) contributed to the gradual peak of shrub species richness and diversity (Zhu et al. 2008; Su et al. 2018), which is consistent with our findings (Figure 8b). The populations of *E. rhytidosperma* on the east side of the Helan Mountains are smaller than those on the west side, with the populations on the eastern and western slopes showing different adaptations to environments (Figure 1a, Figure 8), which could also be related to the degree of anthropogenic disturbances. Long‐term overgrazing on the western slopes of the Helan Mountain has caused the perennial grassland to become scrubby, triggering the enrichment of soil moisture and nutrient heterogeneity (Zheng, He et al. 2008), thus creating suitable environmental conditions for the survival of *E. rhytidosperma* . However, due to increasing land development and utilization in the eastern foothills of the Helan Mountains, large areas of the habitat of *E. rhytidosperma* have been severely decimated (Shi et al. 2022), resulting in increasingly scarce populations.

The spatial distribution pattern of montane biodiversity at small scales is often significantly influenced by its own topography and the microclimatic conditions induced by the surrounding landscape (Yang, Wang et al. 2019). Modeling analysis in this study based on the current climate shows that the precipitation (Bio14) and the temperature (Bio9) are the dominant factors driving the distribution of *E. rhytidosperma* . This is consistent with the findings of Zhang and Tao (2015), which showed that precipitation during the dry season contributed to seed germination of the species. Slope was another major contributing factor in the model prediction, which confirms the conclusion of Zheng, Dong et al. (2008) that slope can regulate soil physicochemical properties, thereby influencing the structure of vegetation communities, and thus encouraging the establishment of *E. rhytidosperma* on steep slopes, rocky outcrops, and thin soil layers. It is worth mentioning that with the gradual climatic changes in the future, the precipitation (Bio14), the temperature (Bio9), and slope will remain dominant factors influencing the geographical distribution pattern of *E. rhytidosperma* (Table 3).

### 4.2 Dynamic Changes of Past and Future Ranges of *E. rhytidosperma*

Since the LGM, global temperature variability has declined by three quarters, followed by gradual stabilization (Rehfeld et al. 2018), which is consistent with the trend in the distribution of *E. rhytidosperma* . Our analyses reveal that the Helan Mountains and surrounding areas have always been an important distribution area for *E. rhytidosperma* since the LIG, despite the large‐scale contraction of suitable environments, and thus we hypothesize that these mountains may have been an ice‐age refugium for *E. rhytidosperma* , as concluded by Bai, Liao, and Zhang (2010).

In addition, the changes in the suitable habitat of *E. rhytidosperma* under various future climatic scenarios are insignificant, with only localized expansion and contraction at the edge of the Helan Mountains (Figure 4). Of these, the three climatic scenarios in 2050 show a decreasing‐increasing trend in suitable habitats, while the three climatic scenarios in 2070 display a decreasing‐increasing‐decreasing trend (Figure 5a). Therefore, the three climatic scenarios predict an eventual decrease in the range during two periods, but still a high suitable area under future climate change, contrary to the conclusions of Lü (2009).

Changes in species distributions can be clearly predicted based on population centroid migration. On a national or global scale, most species tend to migrate long distances with climatic changes (Yan et al. 2021). Our results found that *E. rhytidosperma* has undergone extensive east‐to‐west migration from the LIG to the present, which may be related to the arid events that have occurred in northern China since the Last Glacial period (Lei et al. 2022), forcing the species to migrate inland areas. On the other hand, the population centroid and SDEs of *E. rhytidosperma* under future climatic scenarios would be expected to migrate to the southeast with time, which is contrary to the shifts in the distribution area of Asteraceae (Yang et al. 2023). Coincidentally, *E. rhytidosperma* populations should gradually migrate toward the eastern slopes of the Helan Mountains under future climate changes (Figure 6a), which may be due to an intensification of drought in the west caused by the global warming, forcing the species to migrate toward the wetter eastern regions (Ao et al. 2021; Li et al. 2024).

### 4.3 Conservation Status and Assessment of *E. rhytidosperma*

The results of GeoCAT assessment showed that the EOO and AOO of *E. rhytidosperma* were 1752.82 and 64 km^2^, respectively; in both cases, the endangered status of the species is assessed as Endangered (EN). Based on the results of model simulations under current and future climatic scenarios, we found that the trend of changes in the range of *E. rhytidosperma* was not obvious, and according to the IUCN Red List Standards (IUCN Standards and Petitions Subcommittee 2024), the increase or decrease is lower than the standard value (30%) (Table 6), that is the future habitat of *E. rhytidosperma* is probably in a relatively stable state. Therefore, future climate change will have little impact on conservation planning for this species.

**TABLE 6:** Assessment of the future endangered status of *E. rhytidosperma* .

Period	Change area (×10^4^ km^2^)	Change proportion (%)	Conservation status
Current	0	0	EN
2050 ssp126	−3.532	3.48%	EN
2050 ssp245	−10.826	10.67%	EN
2050 ssp585	−5.299	5.22%	EN
2070 ssp126	−0.152	0.15%	EN
2070 ssp245	+3.838	3.78%	EN
2070 ssp585	−14.872	14.66%	EN

*Note:* The current Red List assessment status of *E. rhytidosperma* is “EN” (endangered), with no significant changes under future climatic conditions, so the original endangered status is maintained.

Species hotspots have been frequently identified as biodiversity conservation priorities (Myers et al. 2000). The results of this study show that the future hotspots of *E. rhytidosperma* will not change much in different periods, and remain concentrated in the Helan Mountains and surrounding areas. However, it is found that only a few hotspots are distributed in nature reserves, creating a large conservation gap. The unprotected areas are mainly located in the marginal areas of the Helan Mountains NNR, which has suffered a serious loss of habitat, increased habitat fragmentation, decreased vegetation cover, and increased ecological risks due to urban expansion, land use, and other anthropogenic activities (Liao et al. 2019; Yang, Yang et al. 2019; Yuan et al. 2023). This could lead to a further decrease in the number and population size of *E. rhytidosperma* . It is important to note that there is still a portion of the hotspot areas where *E. rhytidosperma* is distributed in PNRs, which receive less attention than NNRs in terms of human, financial, and material resources and scientific research (Feng et al. 2020). Moreover, effective conservation measures in these areas will not only prevent the loss of *E. rhytidosperma* populations but also protect other threatened plant populations (e.g., *Tetraena mongolica*, etc.) (Dong et al. 2023).

Unfortunately, the number of NRs in northwestern China is limited and their distribution is discontinuous, with no more continuous NRs having been established (Kuang et al. 2015). However, we found that the hotspots of *E. rhytidosperma* under the current and future climatic scenarios occur in neighboring NRs (Figure 7). In the future, it should be possible to prioritize the NR network in those areas with high abundance in order to improve the connectivity of the neighboring NRs (Saura et al. 2017; Tarabon, Dutoit, and Isselin‐Nondedeu 2021; Wang et al. 2022).

Additionally, we established an *ex situ* database of China gymnosperms in botanical gardens. According to this database, *E. rhytidosperma* is currently only grown in Yinchuan Botanical Garden, so *ex situ* conservation of the species is clearly insufficient. Therefore, we propose to re‐allocate conservation efforts, study the characteristics of population genetics of the species and conduct *ex situ* conservation. The species should be propagated and planted in as many botanical gardens as possible. Moreover, *E. rhytidosperma* possesses superior characteristics such as drought and barren tolerance, and plays an important role in maintaining the stability of desert ecosystems (Zhao et al. 2022). As a result, the species is potentially useful in reforestation of the region. Finally, reproduction of the species has been poorly studied. We observed large numbers of seeds in natural populations, but it remains unclear what the germination ratio of seeds is, and whether the species can reproduce in botanical gardens.

## 5 Conclusion

In this study, we present the first integrative analysis of distribution for *E. rhytidosperma* under past, present, and future climatic scenarios, as well as the first assessment of conservation effectiveness of this species under current and future climatic conditions. The most suitable area for *E. rhytidosperma* at present is in low‐lying parts of the Helan Mountains. The distribution area of *E. hytidosperma* has contracted significantly and migrated westwards since the LIG. The Helan Mountains may have acted as the last refugium for this species. Under future climatic scenarios, the range of the species is expected to fluctuate and will tend to migrate eastwards. Unfortunately, only 15.1% of the actual range of *E. rhytidosperma* is located in nature reserves, indicating a large conservation gap. Effective conservation actions should include both *in situ* and *ex situ* conservation.

## Author Contributions

**Chao Tan:** data curation (lead), investigation (lead), visualization (lead), writing – original draft (lead). **David Kay Ferguson:** writing – review and editing (supporting). **Yong Yang:** conceptualization (lead), investigation (supporting), writing – review and editing (lead).

## Conflicts of Interest

The authors declare no conflicts of interest.

## Data Availability

All data used in the study are included in this paper are available.
